# Chaperonin-Containing TCP1 Subunit 6A Is a Prognostic Potential Biomarker That Correlates With the Presence of Immune Infiltrates in Colorectal Cancer

**DOI:** 10.3389/fgene.2021.629856

**Published:** 2021-05-04

**Authors:** Hui Sun, Yan Wang, Hao-Yu Jing, Xin-Yu Yang, Xin-Xiu Shi, Jia-Hui Zhang, Yuan-Xiu Yu, Li Gao, Xin-Yue Wang, Wan-Hong Li, Lei Yu

**Affiliations:** ^1^College of Pharmacy, Harbin Medical University, Harbin, China; ^2^Pharmaceutical Experiment Teaching Center, College of Pharmacy, Harbin Medical University, Harbin, China; ^3^Department of Colorectal Surgery, The Second Affiliated Hospital of Harbin Medical University, Harbin, China; ^4^Department of Oral and Maxillofacial Surgery, The Second Affiliated Hospital of Harbin Medical University, Harbin, China

**Keywords:** chaperonin-containing TCP1 subunit 6A, colorectal cancer, immune infiltrates, prognosis, functional network analysis

## Abstract

**Aims:**

Chaperonin-containing TCP1 subunit (CCT) 6A is an oncogenic 6th subunit of the CCT family. Nevertheless, not much is documented regarding its function in colorectal cancer (COAD). This investigation seeks to explore the role of *CCT6A* in the prognosis of COAD.

**Main Methods:**

Sequencing data from the Gene Expression Omnibus (GEO) and Cancer Genome Atlas database (TCGA) were employed to analyze the expression of *CCT6A* and its involvement in various regulatory networks behind COAD. Oncomine and Gene Expression Profiling Interactive Analysis (GEPIA) analyzed Levels of expression and survival rates, while GEPIA was used to uncover further the functional networks that involved *CCT6A*. Database for Annotation, Visualization, and Integrated Discovery (DAVID) tools were used to interpret Gene Ontology and Kyoto Encyclopedia of Genes and Genomes pathways. Evaluation of the expression levels of *CCT6A* in COAD samples was also verified via immunohistochemistry.

**Key Findings:**

We found that the expression of *CCT6A* is up-regulated in COAD. *CCT6A* correlated with poor prognosis and decreased immune infiltrates such as CD4^+^ T cells, B cells, and dendritic cells. *CCT6A* is increased in COAD patients. *CCT6A* is associated with several gene networks related to the DDX family and mismatch repair pathways.

**Significance:**

Our data showed that data mining was able to uncover data regarding levels of *CCT6A* and its involvement in genetic regulating pathways in COAD.

## Introduction

COAD is the third most frequently encountered malignancies globally (Brody, 2015). Its incidence and mortality in China have been rising from 2000 to 2011 (Goldstein et al., 2016). There are no apparent symptoms in the early stage of colorectal cancer, with patients often presenting in advanced stages of the disease. Furthermore, symptoms of colorectal cancer manifest across a wide clinical spectrum depending on its primary origin. While advancements in treatment modalities have improved the survival rates of colorectal patients greatly, its prognosis is far from ideal, especially in those with advanced disease (Yu et al., 2018). However, the mechanisms underlying COAD have not been fully understood. Therefore, it is important to clarify COAD’s pathogenesis and uncover new biomarkers that may have therapeutic or prognostic value in patients with colorectal cancer.

Cancer immunotherapy (CI) has proved to be a useful treatment option in those with late-stage colorectal cancer (Zhang et al., 2019). Nevertheless, a small proportion of patients have failed to respond to this therapy, a phenomenon that may be attributed to interactions between the tumor and the immune system (Sarvaria et al., 2017). These immune markers may potentially be a source of prognostic predictors (Wagner and Roth, 2018; Pan et al., 2019). There is evidence that the immune microenvironment is a potential modulator of response to treatment and cancer progression (Dominguez et al., 2019; Leone et al., 2019; Wen et al., 2019). Tumor tissue infiltration by mononuclear immune cells has been reported to occur in several types of solid tumors, including COAD (Liu et al., 2017; Ren and Zhang, 2019). Yin et al. (2017) showed that the immune-microenvironment confers chemoresistance of COAD via IL6. Ye et al. (2019) reported tumor-infiltrating immune cells to play an essential role in COAD prognosis. These evidences highlight immune infiltration as a critical agent in COAD.

The CCT family is known to play oncogene roles in many cancers, especially *CCT6A*. Ma et al. (2018) showed that *CCT6A* was involved in the proteogenomic characterization of human colorectal cancer liver metastasis. Di Meo et al. (2019) reported that *CCT6A* was a potential prognostic biomarker in renal cell carcinoma. Klimczak et al. (2019) also suggested that *CCT6A* may be involved in imparting an unfavorable prognosis in breast cancer tumor progression. Despite this, the involvement of *CCT6A* in COAD has not been clarified.

The current investigation demonstrates the expression of *CCT6A* based on COAD patient data derived from the TCGA and other publicly accessible databases. Clinical data were correlated to the functional genetic networks related to *CCT6A* in COAD by using multi-dimensional analysis methods. A flowchart of our work is shown in Supplementary Figure 1. Our analysis may potentially uncover several novel therapeutic and prognostic treatment targets for COAD management.

## Materials and Methods

### Oncomine Analysis

The Oncomine 4.5 database was used to evaluate *CCT6A* mRNA expression in COAD. Oncomine^1^ is the most significant resource for oncogene chips and functions to host integrated data mining. It currently holds data from 86,733 cancer tissues and normal tissues with 715 gene expression data sets (Rhodes et al., 2007). Our analysis utilized data from a series of COAD studies (at Oct 2020), which included the Notterman colon (28 normal colon tissues vs. 50 COAD tissues), Kaiser colon (5 normal colon tissues vs. 10 COAD tissues), Skrzypczak colorectal (24 normal colon tissues vs. 36 COAD tissues), Ki colon (28 normal colon tissues vs. 50 COAD tissues), Hong colorectal (12 normal colon tissues vs. 70 COAD tissues), and Sabates-Bellver colon (32 normal colon tissues vs. 25 COAD tissues) studies. Detail information of these datasets were shown in Table 1. These datasets were collected and analyzed by Oncomine tool (at Oct 2020). COAD tissues were processed to quantify the *CCT6A* levels concerning its expression in normal tissue. Differences that had a *p*-value of less than 0.01 were considered significant.

**TABLE 1:** The detail information of COAD datasets in our analysis pipeline.

Data name	Data accession	Samples	Usage	Platform
Skrzypczak colorectal dataset	GEO: GSE20916	1. Colorectal Tissue (24)	Differential analysis	Human Genome U133 Plus 2.0 Array
		2. Colorectal Carcinoma (36)		
Ki colon dataset	GEO: GSE6988	1. Colon (28)	Differential analysis	Human 17K cDNA-GeneTrack
		2. Liver (13)		
		3. Colon Adenocarcinoma (50)		
Kaiser colon dataset	GEO: GSE5206	1. Colon (5)	Differential analysis	Human Genome U133 Plus 2.0 Array
		2. Rectosigmoid Adenocarcinoma (10)		
Notterman colon dataset	http://microarray.princeton.edu/oncology/carcinoma.html	0. No value (18)	Differential analysis	Hu6500
		1. Colon Adenocarcinoma (18)		
Hong Colorectal Dataset	GEO: GSE9348	1. Colon (12)	Differential analysis	Human Genome U133 Plus 2.0 Array
		2. Colorectal Carcinoma (70)		
Sabates-Bellver colon dataset	GEO: GSE8671	0. No value (32)	Differential analysis	Human Genome U133 Plus 2.0 Array
		1. Colon Adenoma (25)		
		2. Rectal Adenoma (7)		
Staub et al. colorectal dataset	GEO: GSE12945	1. Colorectal cancer (62)	Survival analysis	Human Genome U133A Array
TCGA colon adenocarcinoma	https://portal.gdc.cancer.gov/	1. Normal Samples (41)	1. Differential analysis	IlluminaHiSeq
		2. Primary Colon Tumor (286)	2. Survival analysis	
		Survival analysis (270)	3. Immune infiltration analysis	

### UALCAN Analysis

UALCAN (v1.0)^2^ is an interactive internet-based platform for carrying out detailed analyses of TCGA genetic expression data and utilizes TCGA 3rd level of clinical and RNA-seq information derived from 31 types of cancer (Chandrashekar et al., 2017). We used UALCAN for quantification of a target gene(s)’ relative expression across different grades of tumors and stages of cancer, across healthy tissue and tumor tissue samples, and several other types of clinicopathological characteristics (at October 2020).

### GEPIA Analysis

GEPIA (v1.0)^3^ is a comprehensive website that allows researchers to perform patient survival analysis, dimensionality reduction analysis, correlation analysis, profiling plotting, differential expression analysis, and detection of similar genes (Tang et al., 2019). The survival analyses of *CCT6A* and its associated COAD genes were performed using this tool (at October 2020).

### TIMER Database Analysis

We utilized the TIMER (v1.0)^4^ database as a resource to analyze the various types of immune infiltrates in COAD (Li et al., 2016). The TIMER database is home to the immune profiles of 10,897 samples across 32 cancer types from TCGA. With this tool, we were able to determine the gene models of the molecular immune profile associated with *CCT6A* expression. Cells included in the analysis were dendritic, macrophage, neutrophil, B cells, CD4^+^ and CD8^+^ T cells (at October 2020).

### Functional Enrichment Analysis

The functional enrichment analysis included the network analysis and Kyoto Encyclopedia of Genes and Genomes (KEGG) pathway and Gene Ontology (GO) enrichment. The STRING online database (v2020)^5^ allowed us to build a protein-protein interaction network (PPI) network consisting of *CCT6A* related genes (Franceschini et al., 2013). DAVID (v6.8) bioinformatics^6^ was implemented in GO KEGG pathway analysis (at Oct 2020). The criteria were set as *P* < 0.05.

### Patients

Eighty pairs of COAD tissue were harvested from patients who received surgery at the Department of Colorectal Cancer Surgery, Second Affiliated Hospital of Harbin Medical University (Harbin, China) between January 2015 and December 2019. The age of the patients was 49 years (range, 28–76 years). Additionally, normal COAD tissue samples were acquired by endoscopy from non-tumor areas from the patients with COAD enrolled in the study. All tissue specimens used in the current study were obtained after getting written informed consent from all participants. Study protocols were reviewed and passed by the Ethics Committee of Harbin Medical University. All the patients’ information was shown in Table 2.

**TABLE 2:** The detail information of COAD patients.

Characteristics	High expression, n (%)	Low expression, n (%)	*P*-value
**Gender**			0.70
Male	25 (59.5)	17 (40.5)	
Female	21 (55.3)	17 (44.7)	
**Age**			0.75
≤60	20 (55.6)	16 (44.4)	
>60	26 (59.1)	18 (40.9)	
**Tumor size (cm)**			0.11
<5	14 (41.2)	20 (58.8)	
≥5	32 (69.6)	14 (30.4)	
**Differentiation**			<0.01
Well + moderately	23 (44.2)	29 (55.8)	
poorly	23 (82.1)	5 (17.9)	
**TNM stage**			0.03
I + II	20 (46.5)	23 (53.5)	
III + IV	26 (70.3)	11 (29.7)	
**Infiltrate depth**			0.13
T1 + T2	5 (38.5)	8 (61.5)	
T3 + T4	41 (61.2)	26 (38.8)	
**Lymph node involvement**			0.05
0	24 (46.2)	28 (53.8)	
≥1	22 (78.6)	6 (21.4)	
**LVIor PNI**			<0.01
Negative	7 (22.6)	24 (77.4)	
Positive	39 (79.6)	10 (20.4)	
**Location**			
Right-side colon	27 (77.1)	8 (22.9)	0.02
Rectum + Left-side colon	19 (42.2)	26 (57.8)	

### Immunohistochemistry (IHC)

IHC was performed following standard procedures as described previously. Anti-CCT6A rabbit polyclonal antibodies (Cat. no. ab191951; 1:100 dilution; Abcam, Cambridge, United Kingdom) was added to the tissue sections before they were left to incubate overnight at 4°C. Following PBS rinse, samples were further incubated with biotinylated secondary antibody (cat. no. 111-035-003; 1:1,500 dilution; Jackson ImmunoResearch, United States) for 30 min at room temperature before being exposed to diaminobenzidine for 5 min at room temperature. The entire experiment was repeated with PBS replacing the primary antibody to produce a negatively staining control specimen.

### Statistical Analysis

The data was a compilation from a minimum of 3 to 6 independent experiments as the mean ± SD (standard deviation). For the clinical tissue test, the data were appraised by a paired Student’s *t*-test, where *P* < 0.05 was indicative of statistical significance. Statistical analyses were performed by SPSS (version 19.0; United States) software and illustrated with GraphPad Prism (version 7.0; United States).

## Results

### *CCT6A* Expression in COAD

**FIGURE 1:**
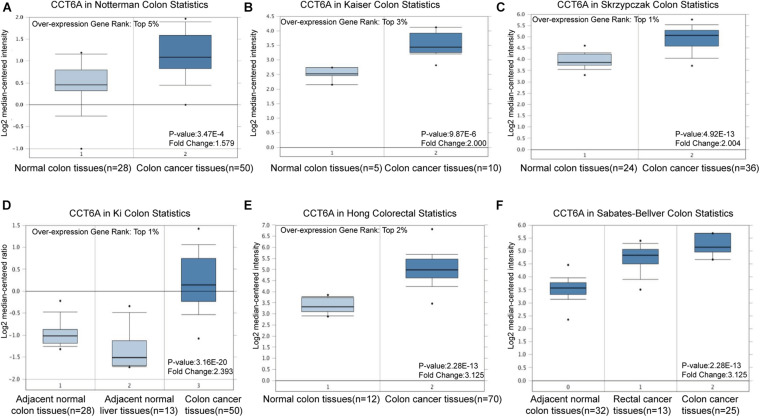
*CCT6A* transcription in COAD (Oncomine). Levels of *CCT6A* mRNA was significantly higher in COAD than that in normal tissue. The fold change, associated *p*-values, and overexpression gene rank, are shown based on Oncomine 4.5 analysis. **(A–F)** The box plot shows CCT6A mRNA levels in the Notterman Colon, Kaiser Colon, Skrzypczak Colon Ki Colon, Hong Colorectal and Sabates-Beliver Colon datasets.

**FIGURE 2:**
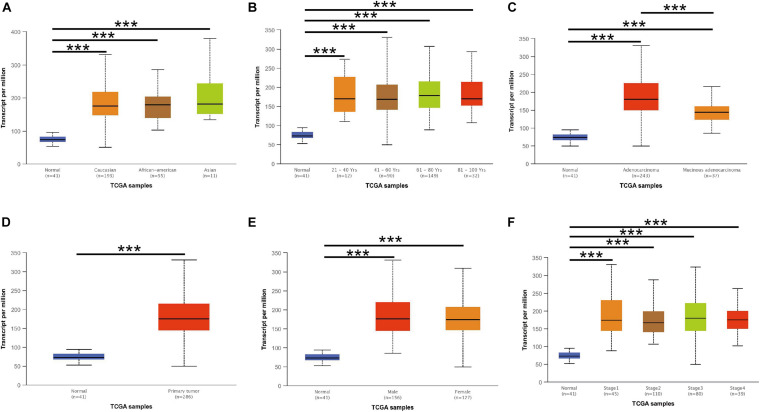
*CCT6A* transcription in subgroups of patients with COAD, stratified based on race, age, and other criteria (UALCAN). **(A)** Boxplot showing relative expression of *CCT6A* in normal and COAD samples. **(B)** Boxplot showing relative expression of *CCT6A* in normal individuals of either gender or male or female COAD patients. **(C)** Boxplot showing relative expression of *CCT6A* in normal individuals of any age or COAD patients aged 21–40, 41–60, 61–80, or 81–100 year. **(D)** Boxplot was showing relative expression of *CCT6A* in normal individuals of any ethnicity or COAD patients of Caucasian, African-American or Asian ethnicity. **(E)** Boxplot showing relative expression of *CCT6A* in normal individuals or COAD patients in stages 1, 2, 3, or 4. **(F)** Boxplot showing relative expression of *CCT6A* in normal individuals or COAD patients with grade 1, 2, 3, or 4 tumors. Data are mean ± SE. ***P < 0.001.

**FIGURE 3:**
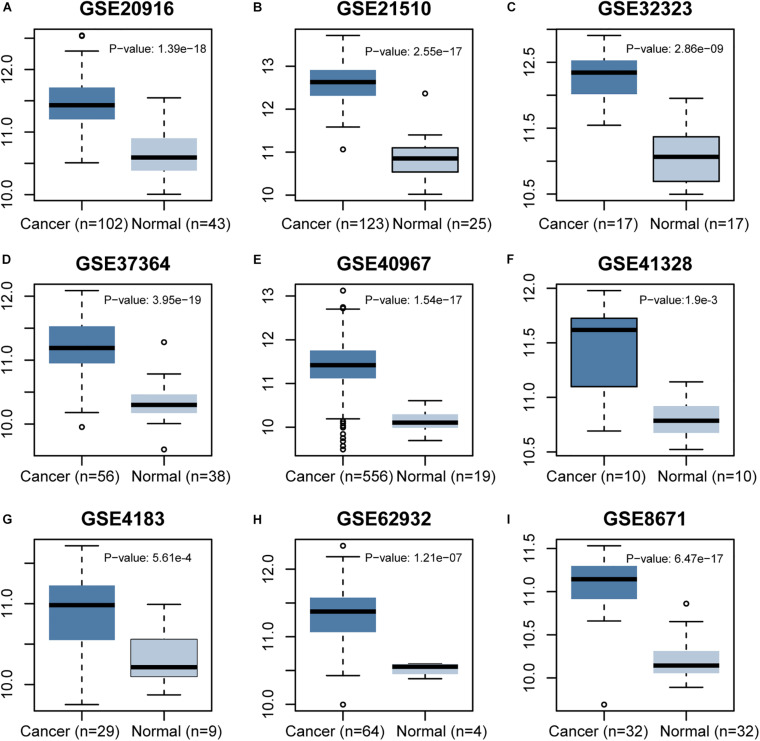
Differential expression analysis of *CCT6A* gene in several COAD datasets of GEO.

**FIGURE 4:**
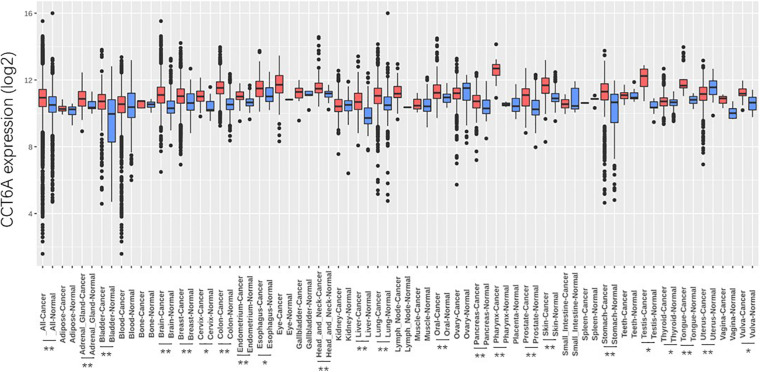
Differential expression analysis of *CCT6A* gene in pan-cancers. Data are mean SE. *P < 0.05 and **P < 0.01.

The *CCT6A* transcription levels across several studies on COAD studies extracted from the GEO database were evaluated. Based on the Oncomine 4.5 database, COAD tissues possessed a markedly raised *CCT6A* mRNA expression profile compared to healthy samples (*p* < 0.01) (Figure 1). The UALCAN database contained sub-group analyses data of COAD based on different clinicopathological shapes and also demonstrated that COAD tissues had higher *CCT6A* levels in contrast to normal samples (Figure 2A). *CCT6A* was differentially expressed in subgroup analyses according to tumor grade, disease stage, histological subtypes, and age, but not gender (Figures 2B–F). We collected more gene expression datasets of COAD from the GEO database, including GSE20916, GSE21510, GSE32323, GSE37364, GSE40967, GSE41328, GSE4183, GSE62932, and GSE8671. Based on these datasets, we have explored the expression variation status of the *CCT6A* gene between COAD and normal tissues. We found the expression levels of *the CCT6A* gene were significantly higher in COAD tissues than in normal tissues (Figures 3A–I). We have also performed differential expression analysis of the *CCT6A* gene in pan-cancers by using the GENET2 (v2.0) web tool^7^ (Park et al., 2019). We found that the *CCT6A* gene was significantly higher in some other cancer tissues than normal tissues (Figure 4). Based on this line of evidence, *CCT6A* may be a potential diagnostic tool in COAD.

### CCT6A Is Up-Regulated in COAD Patients

**FIGURE 5:**
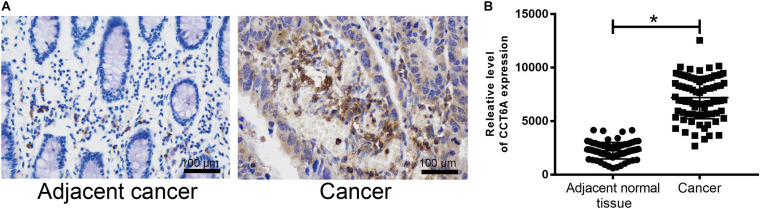
*CCT6A* expression is up-regulated in COAD patients. **(A,B)** Immunohistochemistry was employed to test the protein level of *CCT6A* in COAD patients. Data are mean ± SE. *P < 0.05 vs. normal group.

To determine whether *CCT6A* expression may correlate with COAD, immunohistochemistry was performed. *CCT6A* expression was detected primarily in the cytoplasm in the perinuclear region (Figure 5). According to our data, we found that the protein level of *CCT6A* in COAD is up-regulated in comparison to normal intestinal tissue. Our data suggest that *CCT6A* expression is increased in COAD patients and detected in the cytomembrane.

### High *CCT6A* Expression Impacts the Prognosis of COAD Patients

**FIGURE 6:**
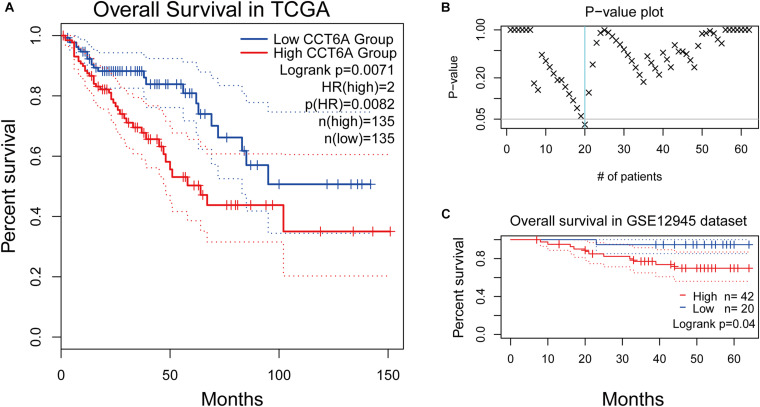
Survival analysis of *CCT6A* in different datasets. **(A)** Kaplan-Meier survival curves comparing the high and low expression of CCT6A in different types of cancer using the GEPIA. **(B)** The *p*-value plot of survival analysis result by ProgScan. **(C)** Kaplan-Meier survival curves comparing the high and low expression of *CCT6A* in the GSE12945 dataset.

The association and impact of *CCT6A* in COAD were further explored by investigating its effect on patient survival. This was performed using the GEPIA databases. A higher level of *CCT6A* correlated with a shorter overall survival time in COAD (*P* = 0.0071), indicating that raised *CCT6A* expressions were a risk factor for poor survival in COAD patients (Figure 6A). Further, we evaluated the prognostic efficiency of *CCT6A* in an independent dataset of 62 COAD cohorts (GSE12945 from the GEO database). Based on this dataset, Kaplan-Meier survival analysis was performed using an online tool, ProgScan, which employs a minimum *P*-value approach to the optimal cut-off point in continuous gene expression (Mizuno et al., 2009). Using this method, we divided the 62 patients into different groups according to the minimum *P*-value and found that *CCT6A* successfully divided patients into different risk groups (Figures 6B,C).

### CCT6A Expression Correlated With Immune-Cell Infiltration in COAD

**FIGURE 7:**
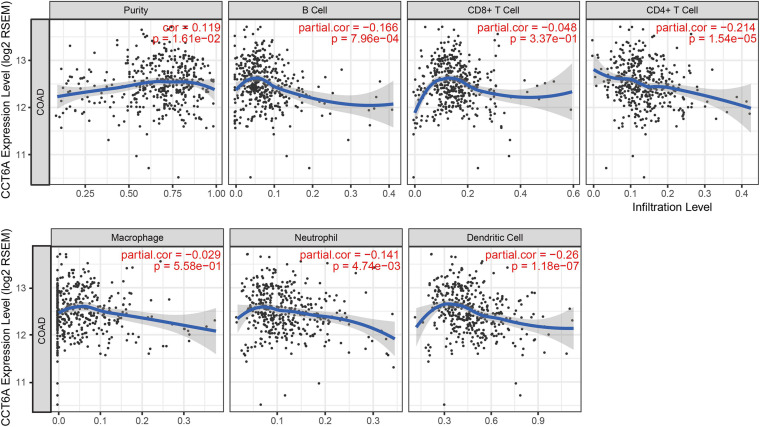
Correlation of *CCT6A* expression with immune infiltration level in COAD (colon adenocarcinoma).

Tumor-infiltrating lymphocytes have been found to have prognostic value in various tumors (Fu et al., 2019). Therefore, we explored if the expression of *CCT6A* was linked to levels of immune infiltrates in COAD using the TIMER resource. We found that *CCT6A* levels correlated negatively with levels of infiltrating B cells, CD4^+^T cells, neutrophils, and dendritic cells but positively associated with tumor purity (Figure 7), suggesting a potentially immune-suppressed role of CCT6A.

### Enrichment Analysis of *CCT6A* Functional Networks in COAD

**FIGURE 8:**
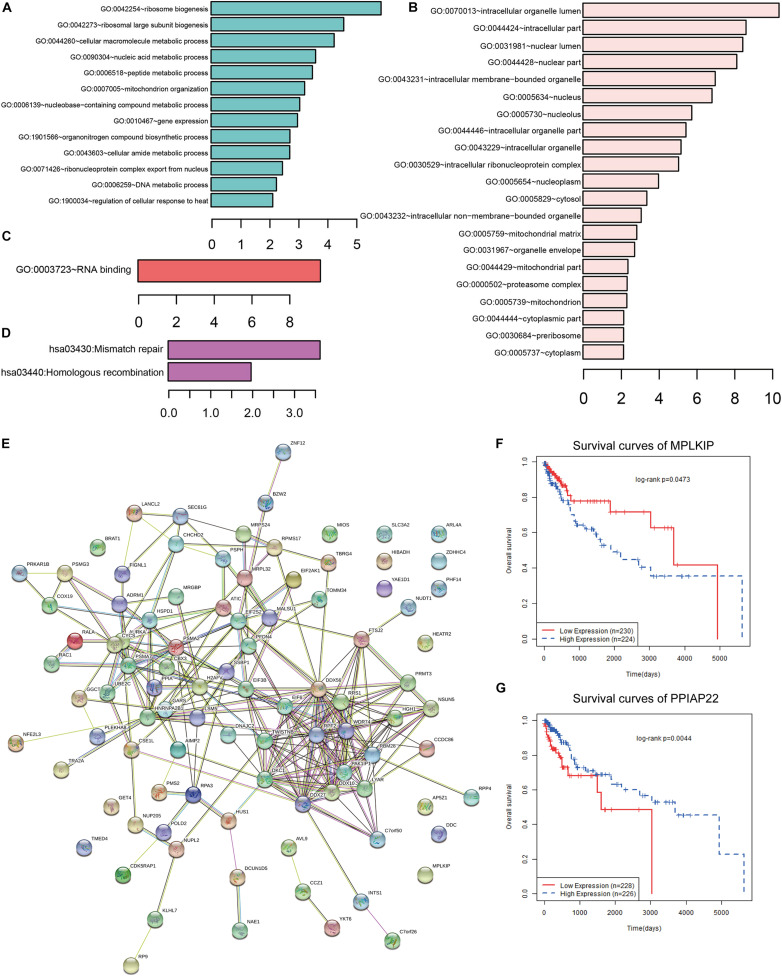
Functional analysis of *CCT6A* in COAD. **(A)** GO BP, **(B)** GO CC, **(C)** GO MF and **(D)** KEGG pathway enrichment analysis of *CCT6A* associated genes. **(E)** The PPI network of *CCT6A* related genes using the STRING online database. **(F,G)** Kaplan-Meier survival curves of *MPLKIP* and *PPIAP22* in COAD patients.

To further investigate the potential target genes of *CCT6A* in COAD, we identified the top 100 associated genes of *CCT6A* in COAD using the GEPIA database. We uploaded all the 100 genes to DAVID to determine overrepresented KEGG pathways and GO categories. Data analysis with GO demonstrated that the following biological processes (BP) had enriched genes: ribosome biogenesis and cellular macromolecule metabolic process (Figure 8A and Table 3). For cell component (CC), the up-regulated DEGs were increased in intracellular organelle lumen and intracellular part (Figure 8B). Furthermore, molecular function (MF) analysis also indicated enrichment in RNA binding (Figure 8C). Figure 8D contains the most significantly enriched KEGG pathways of these genes, including Mismatch repair and Homologous recombination.

**TABLE 3:** The detail information of tools used in our analysis pipeline.

Tool name	Version	Description	Accession
Oncomine	v4.5	A tool for oncogene chips data collection and integrated data mining	http://www.oncomine.org
UALCAN	v1.0	An online platform to perform detailed analyses of genetic expression data on level of clinical and RNA-seq information of TCGA datasets	http://ualcan.path.uab.edu
GEPIA	v1.0	A comprehensive website that allows for researchers to perform patient survival analysis, dimensionality reduction analysis, correlation analysis, etc.	http://gepia.cancer-pku.cn/
TIMER	v1.0	A web server allows users to input function-specific parameters, with resulting figures dynamically displayed to conveniently access the tumor immunological, clinical, and genomic features	https://cistrome.shinyapps.io/timer/
DAVID	v6.8	A tool provides a comprehensive set of functional annotation tools for investigators to understand biological meaning behind large list of genes	https://david.ncifcrf.gov/
GENET	v2.0	A tool to explore gene expression patterns across different normal and tumor tissues compiled from public gene expression data sets	http://gent2.appex.kr/gent2/

Using the STRING online database (see text footnote 5), 95 out of the 100 *CCT6A* associated genes were screened into the PPI network complex (Figure 8E). Some network nodes were of a higher degree and displayed hubs features (For example, *DDX27*, *DDX56*, *DDX10*, and *RPF2*). We also found that several genes such as *MPLKIP* and *PPIAP22* in this network were significantly associating with COAD patients’ survival (Figures 8F,G).

## Discussion

COAD is the most common intestinal tumor worldwide, especially in women (Hassanlou et al., 2019), and has been reported to be associated with many oncogenes (Yin et al., 2017). Existing literature reports *CCT6A* to possess oncogenic features in a various human cancer (Di Meo et al., 2019; Huang et al., 2019). This study of bioinformatics analysis of publically available genetic sequencing data was performed to gain further insight into the role of *CCT6A* in the genetic regulation of COAD. We used a panel of bioinformatics tools to perform systematic analysis of *CCT6A* across different COAD datasets. Oncomine is the biggest resource and tool for oncogene chips data collection and integrated data mining. It currently holds data from 86,733 cancer tissues and normal tissues with a total of 715 gene expression data sets. UALCAN is an online platform to perform detailed analyses of gene expression data on the level of clinical and RNA-seq information of TCGA datasets. TIMER web server is a comprehensive resource for systematical analysis of immune infiltrates across diverse cancer types. TIMER web server allows users to input function-specific parameters, with resulting figures dynamically displayed to conveniently access the tumor immunological, clinical, and genomic features. David provides a comprehensive set of functional annotation tools for investigators to understand biological meaning behind large list of genes. Transcriptional sequencing data derived from hundreds of clinical samples from TCGA and GEO revealed that COAD tissues possessed higher *CCT6A* mRNA levels in contrast to healthy colon tissue (Figures 1–4).

To ensure if the level of *CCT6A* correlates from the onset of COAD. Immunohistochemistry analyses were employed to test the expression of CCT6A in COAD patients. Our data indicate that *CCT6A* was up-regulated in COAD patients and detected in the cytomembrane (Figure 5). These results suggested that aberrantly increased *CCT6A* levels are a feature of COAD, and may benefit from clinical validation studies to determine its value as a potential prognostic biomarker. There was a consistent correlation between *CCT6A* expression and patient prognosis, indicating that higher levels of this gene were indicative of poorer prognosis (Figure 6A). Based on an independent COAD dataset, *CCT6A* could also successfully divided patients into different risk groups (Figures 6B,C).

This study also sought to clarify the association between tumor immune cell infiltrates and *CCT6A* expression in COAD. Lower *CCT6A* expression was associated with higher infiltrating B cells, CD4^+^ T cells, neutrophils, and dendritic cells, but positively correlated with tumor purity (Figure 7). A significant proportion of immune cell infiltrated in COAD constituted of B cells, specifically terminally differentiated memory B cells or plasma cells, and highlights the presence of a specific immune response in this condition (Shimabukuro-Vornhagen et al., 2014). In the early stages of Crohn’s-like lymphoid reaction development, there is clustering of mature antigen presenting dendritic cells and CD4^+^ T-cells. As Crohn’s-like lymphoid reaction matures, lymphoid follicles are created as a result of increased recruitment of B-cells, as well as follicular dendritic cells (Maoz et al., 2019). Increased macrophage infiltration in colorectal cancer tissues was significantly associated with increased chemoresistance and poor prognosis (Yin et al., 2017). Patients who underwent resection of colorectal cancer have been reported to possess a novel neutrophil phenotype comprising increased phagocytosis, less apoptosis, and lowered NET formation (Richardson et al., 2017)Dendritic cells’ presence dictates a tumor’s response to immunotherapy, effector T cell trafficking to the tumor site, and T cell anti-tumor immunity (Hope et al., 2017). When interpreted as a whole, *CCT6A* may possess a role in polarization of tumor-associated macrophages (TAM).

To further gain insight into *CCT6A* regulated network, we identified the top 100 related genes of *CCT6A* in COAD using the GEPIA database. We constructed a PPI network of these related genes using the STRING database. The results showed that the DDX27, DDX10, and DDX56 were hub genes of the *CCT6A* regulated network. *DDX* (DEAD-box helicase) is the biggest RNA helicase family responsible for short RNA duplex unwinding, a key regulator of RNA biogenesis. Human *DDX3X* and its yeast ortholog Ded1p are a DDX related closely to the human *DDX4* and fly Vasa subfamily. As with all other DDXs, Like all DDXs, *DDX3X* comprises two RecA-like domains (D1D2) which makes up its helicase core. It contains 12 highly conserved sequence motifs (Figure 1A). The D1D2 core of DDX3X is flanked by largely unstructured N- or C-terminal tails, but have motifs responsible for the subfamily’s unique functions. Yang C et al. showed that *DDX27* possessed an oncogenic role in COAD by regulating the stem cell-like activity of COAD cells (Liu et al., 2019). Shi et al. (2019) suggested that higher *DDX10* expression was deletion inhibiting key cellular activities by MAPK signaling pathway (Shi and Hao, 2019). Kouyama et al. (2019) reported DDX56 regulated COAD cell proliferation and cell cycle both *in vitro* and *in vivo* by promoting WEE1. Pathway and GO enrichment analysis revealed that the top 100 related genes of *CCT6A* were mainly related to ribosome biogenesis and mismatch repair (Figures 8A–D). We also found that several genes such as *MPLKIP* and *PPIAP22* in this network were significantly associating with COAD patients’ survival (Figures 8F,G).

In summary, *CCT6A* mRNA levels were markedly raised in COAD in contrast to normal colon tissue and correlates with poor prognosis. We also found that expression level of *CCT6A* is related to decreased immune infiltrates of B, CD4^+^ T and dendritic cells. Finally, the correlated network and functional annotation analysis revealed that *CCT6A* was related to the DDX family. This study provides several supporting lines of evidence that highlight the critical role of *CCT6A* in COAD and its potential as a prognostic marker.

## Data Availability Statement

The raw data supporting the conclusions of this article will be made available by the authors, without undue reservation.

## Ethics Statement

Study protocols were reviewed and passed by the Ethics Committee of Harbin Medical University (KY-2016-036). The patients/participants provided their written informed consent to participate in this study.

## Author Contributions

HS and YW: conception and design. YW, HS, and H-YJ: administrative support. X-YY, X-XS, J-HZ, Y-XY, and LY: provision of study materials. YW, HS, LG, and LY: collection and assembly of data. H-YJ, X-YY, X-XS, J-HZ, Y-XY, LG, and LY: data analysis and interpretation. All authors: manuscript writing and final approval of manuscript.

## Conflict of Interest

The authors declare that the research was conducted in the absence of any commercial or financial relationships that could be construed as a potential conflict of interest.

## Footnotes

1 www.oncomine.org

2 http://ualcan.path.uab.edu

3 http://gepia.cancer-pku.cn/

4 https://cistrome.shinyapps.io/timer/

5 http://string-db.org

6 https://david.ncifcrf.gov/

7 http://gent2.appex.kr/gent2/

## Supplementary Material

Supplementary Figure 1 The flow chart of our analysis steps.
