# Supplementary figures and images for: Chaperonin-Containing TCP1 Subunit 6A Is a Prognostic Potential Biomarker That Correlates With the Presence of Immune Infiltrates in Colorectal Cancer
Source: Front Genet. 2021 May 4;12:629856. doi: 10.3389/fgene.2021.629856 (PMC8129517; doi:10.3389/fgene.2021.629856)

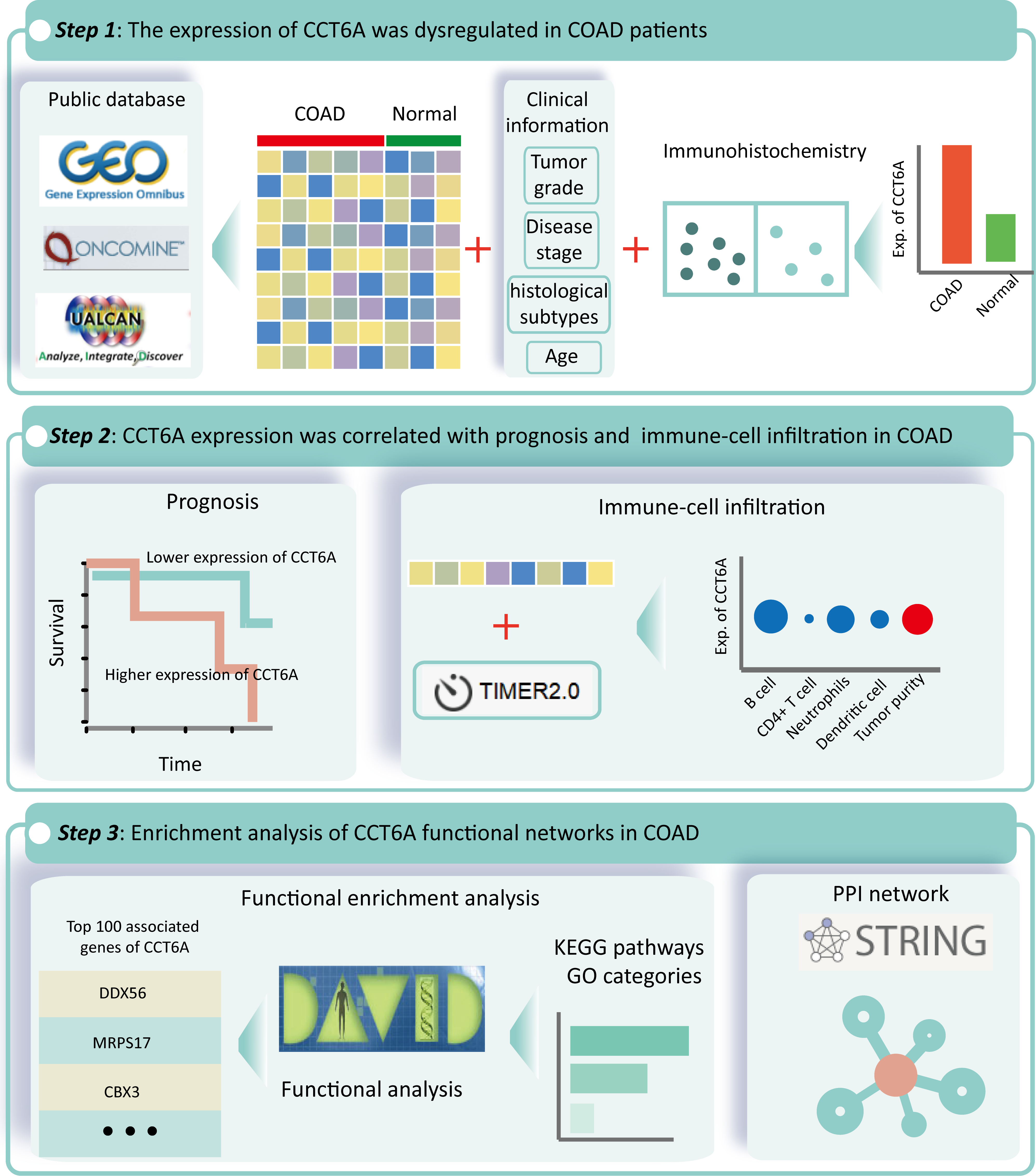

Supplement: Supplementary Figure 1 — The flow chart of our analysis steps. [file Image_1.TIF]
